# Fabrication of Submicrometer-Sized Meloxicam Particles Using Femtosecond Laser Ablation in Gas and Liquid Environments

**DOI:** 10.3390/nano11040996

**Published:** 2021-04-13

**Authors:** Eszter Nagy, Attila Andrásik, Tamás Smausz, Tibor Ajtai, Fruzsina Kun-Szabó, Judit Kopniczky, Zoltán Bozóki, Piroska Szabó-Révész, Rita Ambrus, Béla Hopp

**Affiliations:** 1 Department of Optics and Quantum Electronics, University of Szeged, Dóm tér 9, 6720 Szeged, Hungary; andrasika@titan.physx.u-szeged.hu (A.A.); tomi@physx.u-szeged.hu (T.S.); ajtai@titan.physx.u-szeged.hu (T.A.); kszfruzsina@titan.physx.u-szeged.hu (F.K.-S.); jkopniczky@titan.physx.u-szeged.hu (J.K.); zbozoki@physx.u-szeged.hu (Z.B.); bhopp@physx.u-szeged.hu (B.H.); 2 Institute of Pharmaceutical Technology and Regulatory Affairs, University of Szeged, Eötvös utca 6, 6720 Szeged, Hungary; revesz@pharm.u-szeged.hu (P.S.-R.); ambrus.rita@szte.hu (R.A.); 3 Interdisciplinary Excellence Centre, Department of Materials Science, University of Szeged, Dugonics tér 13, 6720 Szeged, Hungary

**Keywords:** poorly water-soluble drug(s), preformulation, particle size, nanoparticle(s), nanosphere(s)

## Abstract

In pharmaceutical development, more and more drugs are classified as poorly water-soluble or insoluble. Particle size reduction is a common way to fight this trend by improving dissolution rate, transport characteristics and bioavailability. Pulsed laser ablation is a ground-breaking technique of drug particle generation in the nano- and micrometer size range. Meloxicam, a commonly used nonsteroidal anti-inflammatory drug with poor water solubility, was chosen as the model drug. The pastille pressed meloxicam targets were irradiated by a Ti:sapphire laser (τ = 135 fs, λ_c_ = 800 nm) in air and in distilled water. Fourier transform infrared and Raman spectroscopies were used for chemical characterization and scanning electron microscopy to determine morphology and size. Additional particle size studies were performed using a scanning mobility particle sizer. Our experiments demonstrated that significant particle size reduction can be achieved with laser ablation both in air and in distilled water without any chemical change of meloxicam. The size of the ablated particles (~50 nm to a few microns) is approximately at least one-tenth of the size (~10–50 micron) of commercially available meloxicam crystals. Furthermore, nanoaggregate formation was described during pulsed laser ablation in air, which was scarcely studied for drug/organic molecules before.

## 1. Introduction

Laser ablation is a widely used and versatile technique, especially in material processing. It can be applied to produce high-precision surface structures as well as submicrometer-sized particles from various materials in vacuum, gas or liquid. Nanoparticle formation by laser ablation has been extensively investigated in terms of the influence of laser parameters, target properties and ambient conditions. Whereas previous research mainly focused on metals [1,2,3,4,5] and metal alloys [6,7,8], in the past decade more and more studies have been published on particle generation by the laser ablation of organic materials too [9,10,11,12,13].

In the field of pharmaceutical technology, there is increasing demand for the production of nano- and microparticles of drugs. Almost half of the currently marketed drugs are poorly water-soluble, and this ratio is gradually increasing, since 90% of drugs are characterized as poorly water-soluble in the development phase [14]. Particle size reduction (and thereby improvement in the surface to volume ratio) is a possible way to enhance the dissolution rate, transport characteristics and bioavailability of these drugs [15,16]. Size reduction can be achieved by different approaches [17,18], such as forming solid dispersions [19], grinding [20], wet milling [21], cavitation [22], and laser ablation [11,12,13,23,24,25,26,27,28,29]. Laser ablation as a simple, rapid, easily adjustable and chemical-free method has received considerable attention in drug preformulation. It allows the production of submicron-sized particles without inducing any chemical damage of the drugs. Ablation can be implemented in a vacuum/gas chamber or under liquid.

Meloxicam is a poorly water-soluble drug (its solubility in water is 7.15 mg/L at 25 °C [30]) classified as a nonsteroidal anti-inflammatory drug (NSAID) usually prescribed to treat pain, rheumatoid arthritis, osteoarthritis and other joint diseases [31]. Our aim is to compare the ablation of a pure meloxicam target with femtosecond laser pulses in air and in distilled water, referred to as pulsed laser ablation (PLA) and pulsed laser ablation in liquid (PLAL), respectively, in this paper. To find the experimental parameters where chemical damage to meloxicam can be avoided, we examine the morphology and size of the produced particles. Additionally, we give an incipient description of the underlying processes and compare them during PLA and PLAL.

## 2. Materials and Methods

### 2.1. Meloxicam

Meloxicam (4-hydroxy-2-methyl-N-(5-methyl-2-thiazolyl)2H-benzothiazine-3-car-boxamide-1,1-dioxide) obtained from EGIS Ltd., (Budapest, Hungary) is a yellow, 100% crystalline powder with an average particle size of 10–50 µm.

### 2.2. Laser Source

The TeWaTi laser system of the University of Szeged containing a Ti:sapphire-based multipass CPA (chirped pulse amplification) amplifier seeded by a mode-locked laser oscillator (Spectra-Physics Rainbow™ CEP4 provided by Spectra-Physics 3635 Peterson Way, Santa Clara, CA, USA) provided amplified 135 fs pulses at a central wavelength of 800 nm with adjustable energies up to 1 mJ and a repetition rate between 1 and 10 Hz. An optical shutter (Thorlabs Inc. SH05-Optical Beam Shutter with 10′ Long Cable, Ø1/2″ Aperture, 8-32 Taps provided by Thorlabs Inc., Newton, NJ, USA) with a benchtop shutter controller (Thorlabs Inc. SC10-Optical Beam Shutter Controller provided by Thorlabs Inc., Newton, NJ, USA) was used at the output of the amplifier to select a given number of pulses from the train.

### 2.3. Target

The targets were pastille pressed from 0.3 g commercially available meloxicam powder by a hydraulic compactor at 175 MPa pressure.

### 2.4. Pulsed Laser Ablation in Air (PLA)

**Figure 1:**
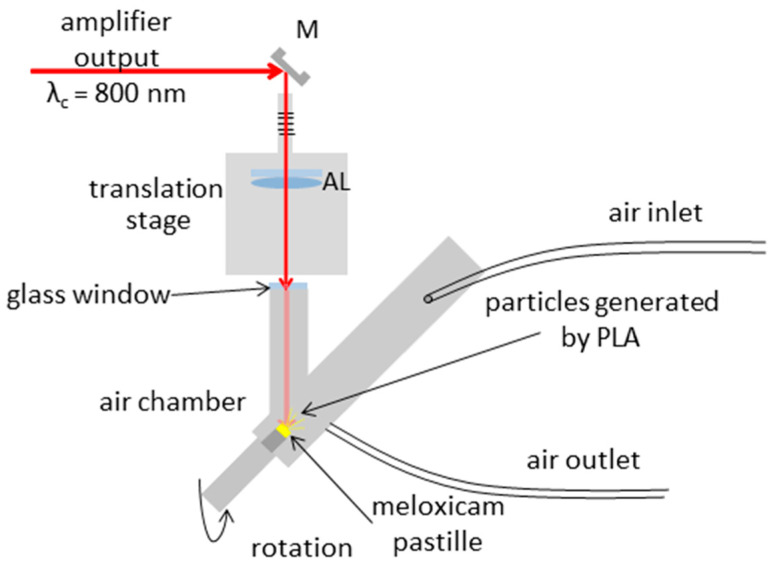
Experimental setup for pulsed laser ablation in air. M: silver mirror, AL: achromatic lens.

The PLA experimental setup is sketched in Figure 1. Pulses provided by the laser amplifier are reflected from a silver plane mirror (M) and propagated through an achromatic lens (AL; focal length of 150 mm) enabling focusing onto the target. The lens is placed on a stage to ensure precise spot size control on the target surface. The input window of the air chamber is a thin piece of glass placed at normal incidence. A meloxicam pastille was placed into the air chamber at a 45° incident angle, with its backside taped to the sample holder platform, right in front of the focal plane of the lens. The sample was rotated in order to avoid fatal degradation during processing. Air flow was pumped through the chamber (maintaining normal pressure) to deliver the generated particles to a scanning mobility particle sizer (SMPS) for size distribution measurements or to a filter collecting the particles for Fourier transform infrared (FTIR) and Raman spectroscopy measurements.

The laser spot size on the target surface, determined by measuring the horizontal dimensions of the ablated holes by optical microscopy, was found to be 111 and 157 µm, denoting the 1/e^2^ beam radii along the minor and major axes, respectively. Pulse energy values were recorded by an energy meter. The applied average laser fluence reaching the target was varied between 0.7 and 1.5 J/cm^2^ by adjusting the output energy of the amplifier.

### 2.5. Pulsed Laser Ablation in Liquid (PLAL)

**Figure 2:**
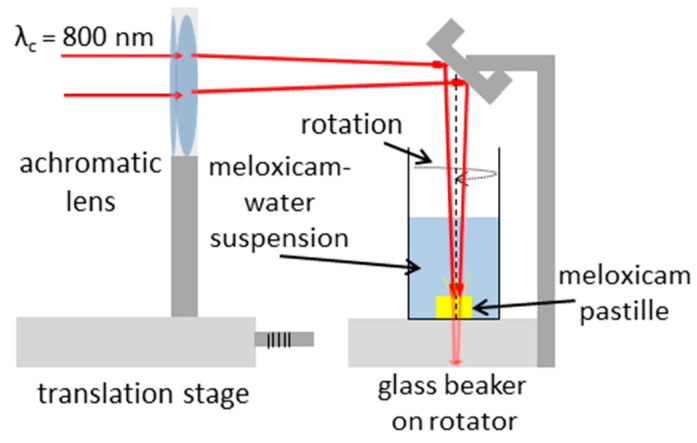
Experimental setup for pulsed laser ablation in liquid.

The PLAL experimental setup is sketched in Figure 2. The amplified beam propagated through an achromatic lens (AL) with a focal length of 150 mm, which focused the beam into the bulk of the target placed at the bottom of a 100 mL glass beaker filled with water. The pastille surface was approximately 5 mm below the water surface. The sample was irradiated at normal incidence, and the circular laser spots on the surface had similar areas as in the PLA experiments.

During target processing, 72,000 or 108,000 pulses were shot onto the surface with a repetition rate of 10 Hz. The sample pastille was taken out of the beaker after laser processing, and the suspension was further investigated.

### 2.6. Fourier Transform Infrared Spectroscopy (FTIR)

In the case of PLA, the generated particles were removed by air flow and collected on a teflon filter (pore size: 1 µm) for chemical analysis. Ablation was carried out until a layer was formed on the filter and the pores were clogged, so particles under the pore size were captured too. A few mg of the particles forming the layer was mixed with 150 mg KBr. The mixture was ground in an achate mortar and pressed into a disk with 10 kN force for Fourier transform infrared (FTIR) analysis.

In the case of PLAL, first, the water was evaporated at around 50 °C from the obtained suspension. The samples were placed into a preheated laboratory oven (CARBOLITE 2416, Thermal Engineering Services Ltd., Worcester, England) for 12 h at 50 °C, and then the dried powder was used for FTIR sample preparation as before.

FTIR spectra were recorded with an FTIR spectrometer (Thermo Nicolet AVATAR 330, LabX Midland, ON, Canada) between 4000 and 400 cm^−1^, at a resolution of 4 cm^−1^, averaging 128 scans/measurement with baseline correction.

### 2.7. Raman Spectroscopy

The samples for Raman spectroscopy were the same as the samples for FTIR. In the case of PLA, the particles were captured on a teflon filter, and in the case of PLAL the dried powders were investigated.

Raman spectra were acquired via a Thermo Scientific™ DXR™ Raman microscope (Thermo Fisher Scientific Inc., Waltham, MA, USA), using λ = 780 nm laser radiation for excitation. The laser power was 2 mW and the estimated spot size at the sample was approximately 1 μm. Raman spectra were recorded between 2500 and 500 cm^−1^ using a 400 lines/mm grating. The resolution was 4.7–8.7 cm^−1^. Each spectrum was recorded by scanning 20 times with a 2 s integration time.

### 2.8. Scanning Electron Microscopy (SEM)

We studied morphology and particle size with scanning electron microscopy (SEM) using a Hitachi S-4700 SEM system (Hitachi S4700, Hitachi Scientific Ltd., Tokyo, Japan) at 10 kV and 10 µA. Prior to SEM, we coated the samples with gold or gold–palladium alloy using a sputter-coater (Bio-Rad SC 502, VG Microtech, Uckfield, UK).

In the case of PLA, a small piece of the particle-collecting filter was preserved as a sample for SEM investigations.

In case of PLAL, after ablation the suspension was stirred up and a droplet was taken from the middle. The specimen droplet was placed on a silicone plate and left to dry before being sputter-coated and imaged by SEM.

### 2.9. Scanning Mobility Particle Sizer (SMPS)

The size distribution of PLA-generated particles was determined with a scanning mobility particle sizer (SMPS, equipped with a Vienna-type DMA + CPC, Grimm Aerosol Technik GmbH & CO. KG, Ainring, Germany with a size range of 10.1–1093 nm).

A scan series by SMPS contains 3 or 4 consecutive measurements, and each scan takes 7 min. During the process, the repetition rate of the laser was 1 Hz and the flow rate of the purging gas was 0.3 L/min.

## 3. Results

### 3.1. Chemical Composition (FTIR, Raman)

**Figure 3:**
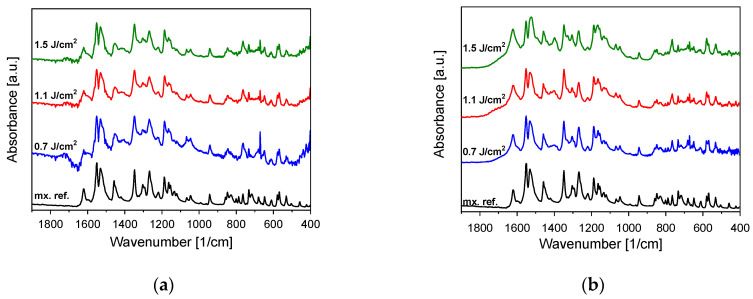
(**a**) Fingerprint part of the Fourier transform infrared (FTIR) spectra of pulsed laser ablation (PLA)-generated particles and the original meloxicam (mx. ref.) powder as reference. (**b**) Fingerprint part of the FTIR spectra of pulsed laser ablation in liquid (PLAL)-generated particles and the original meloxicam (mx. ref.) powder as reference. The FTIR spectra were normalized in both cases to the peak at around 1550 cm^−1^.

FTIR studies of particles generated by PLA and PLAL both show a close resemblance to the original meloxicam (Figure 3). The characteristic peaks appear at the expected wavenumbers. Apparently, there are no tendencies towards decreasing fluence in the investigated range. Nevertheless, minor changes can be discovered in the relative sizes of some peaks (e.g., around 1400 cm^−1^), and in the case of smaller fluences the signal to noise ratio decreases due to the smaller amount of generated particles.

**Figure 4:**
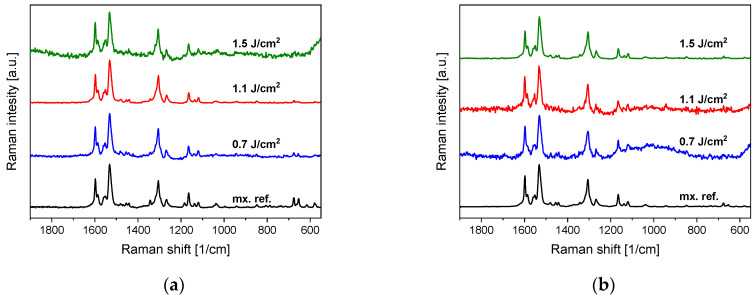
(**a**) Raman spectra of PLA-generated particles and the original meloxicam (mx. ref.) powder as reference. (**b**) Raman spectra of PLAL-generated particles and the original meloxicam (mx. ref.) powder as reference. The Raman spectra were normalized in both cases to the peak at around 1530 cm^−1^.

Raman spectra were recorded from several places in each sample to provide an overview of spatial repeatability. The Raman spectra of both the PLA- and PLAL-generated particles match the Raman spectra of crystalline meloxicam (Figure 4). Therefore, Raman spectroscopy confirms the FTIR observation that no chemical changes occurred during or after the ablation process.

### 3.2. Particle Size Comparison (SEM, SMPS)

#### 3.2.1. SEM

We used SEM imaging for the morphological studies. The images of PLA- and PLAL-generated particles and ablation tracks are a representative selection of the almost 200 recorded images.

**Figure 5:**
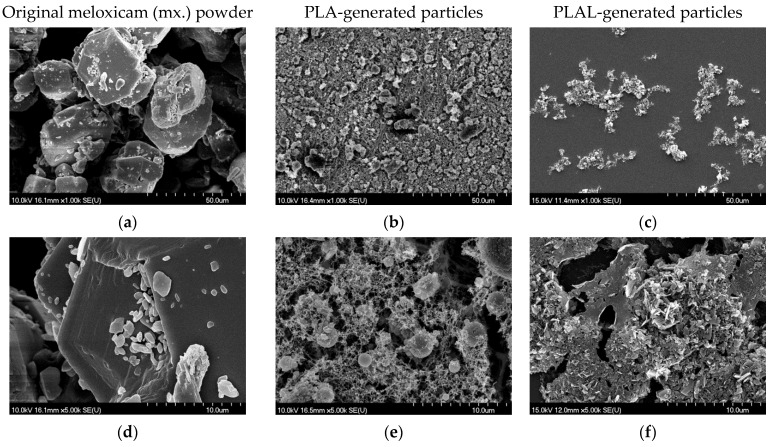
Scanning electron microscopy (SEM) pictures of (**a**,**d**) the original meloxicam powder, (**b**,**e**) the PLA-generated particles collected on a teflon filter and (**c**,**f**) the PLAL-generated particles placed on a silicon plate. Magnification was 1k and 5k in the upper (**a**–**c**) and lower (**d**–**f**) row, respectively.

The size reduction of both PLA- and PLAL-generated particles was clearly visible in comparison to the original powder (Figure 5). We assume that the different morphologies are due to the different particle formation mechanisms during PLA and PLAL.

**Figure 6:**
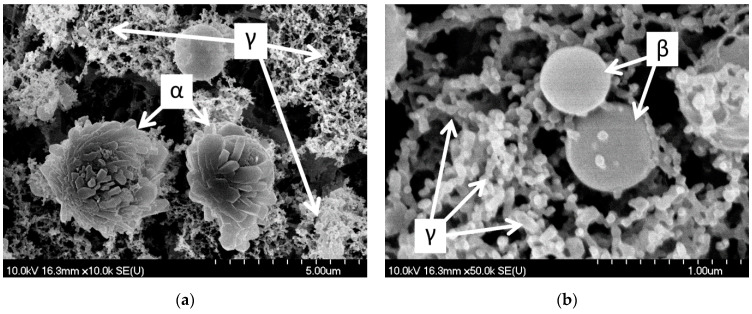
SEM pictures of PLA-generated particles (at 1.5 J/cm^2^ fluence) collected on the filter: (α) spheres with an irregular surface covered by laminar blocks; (β) perfectly smooth spheres and (γ) small (~0.1 µm in diameter) spherical particles joined into chains, creating a web-like structure. (**a**) M: 10k; (**b**) M: 50k.

The shapes of PLA-generated particles can be classified into three groups: (α) spheres with an irregular surface (these particles seem to have a sphere core (~2 µm in diameter) covered by laminar blocks (about 1 µm × 0.5 µm × 0.25 µm)); (β) perfectly smooth spheres (~0.5–1.0 µm in diameter) and (γ) very small (~0.1 µm in diameter) spherical particles joined into chains, creating a web-like structure, as can be seen in the higher-magnification SEM images (Figure 6).

**Figure 7:**
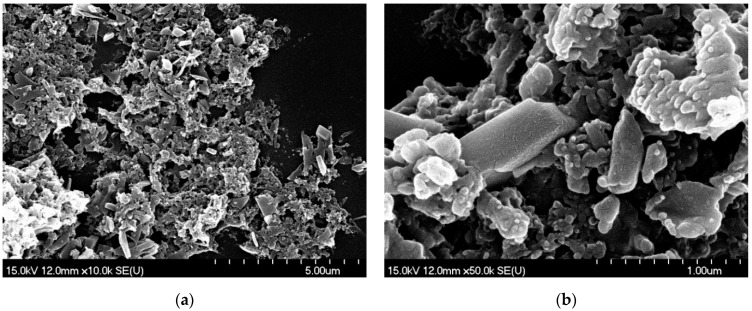
SEM pictures of PLAL-generated particles (at 1.5 J/cm^2^ fluence) dried on a silicon plate: crystal-like particles as well as near-amorphous particles and a shapeless mass of conglomerated material. (**a**) M: 10k; (**b**) M: 50k.

The particles created by PLAL formed small piles during the drying of the droplet. Most of the particles resembled broken glass pieces with sharp edges and smooth surfaces, which can be a result of mechanical effects (Figure 7). Moreover, near-amorphous particles and a shapeless mass of conglomerated material in the background were also seen.

**Figure 8:**
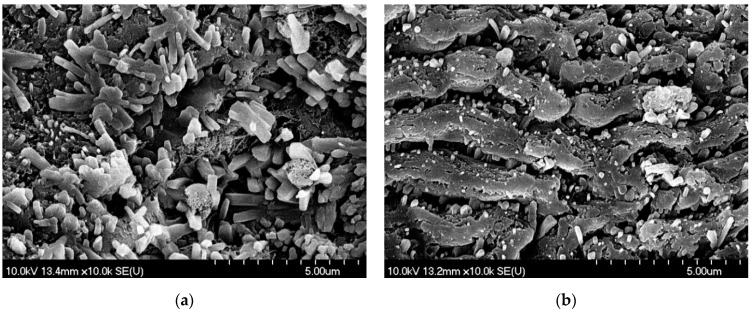
SEM pictures of the meloxicam pastille surface after PLA (at 1.5 J/cm^2^ fluence): (**a**) random or flower-shaped, block-like structures; (**b**) parallel lines with approximately 1.8 µm period length. M: 10k.

To obtain information about the particle formation mechanism during PLA/PLAL, we also investigated the ablated surface of the pastille. In the case of PLA, we observed random or flower-shaped, block-like structures (Figure 8). It must be noted that parts of the ablated tablets were covered with parallel lines with approximately 1.8 µm period length, exhibiting a close resemblance to laser-induced periodic surface structure (LIPSS) formation, which often occurs as a result of the femtosecond ablation of different materials [32].

**Figure 9:**
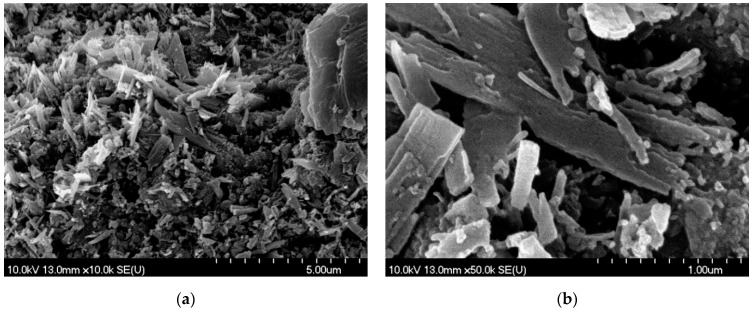
SEM pictures of the meloxicam pastille surface after PLAL (at 1.5 J/cm^2^ fluence): (**a**) the ablated surface of the pastille covered with needle-like and laminar particles. M: 10k; (**b**) magnified central region of (**a**). M: 50k.

In the case of PLAL, the ablated surface of the pastille was covered with needle-like and laminar particles, suggesting mechanical fragmentation (Figure 9).

#### 3.2.2. SMPS PLA

**Figure 10:**
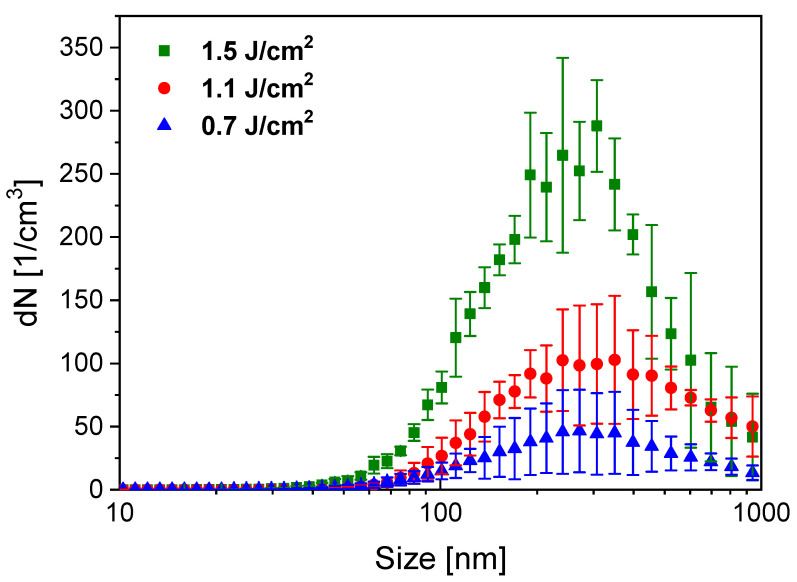
Size distribution of PLA-generated particles for different applied laser fluences.

Size distributions of PLA-generated particles are shown in Figure 10. According to the SMPS data, particle yield increases with higher fluence, while the shape and the maximum of distribution do not show any fluence dependence in the investigated range. We can also confirm that PLA can significantly reduce the original size of the meloxicam particles (10–50 µm). Although the size distribution of the ablated particles is wide (60 nm to ~1 µm), we can conclude that the average particle size is around 200–300 nm. The tail in the size distribution curve around 1 µm can be attributed to particle overcharging (a particularity of the SMPS detection method), which causes a statistical error, increasing the number of detected large particles. Therefore, it is reasonable to omit the SMPS detection of bigger particles.

## 4. Discussion

Particle size reduction of meloxicam was successfully achieved by PLA and PLAL. FTIR and Raman analyses confirmed that no significant changes had occurred in the chemical structure of the drug molecules during the laser treatments. However, the morphologies of the generated particles showed significant differences after ablation in air or in water, which implies the possibility of different mechanisms.

During the interpretation of the PLA results, it must be noted that the size and morphology of the generated particles evolve with time. Therefore, the particles measured in situ by SMPS and those that were imaged later by SEM can be different. Nevertheless, these two methods can give complementary information regarding the pharmaceutical application possibilities of ablated drug particles. SMPS measurements reflect the aerodynamic characteristics of the particles/aggregates, mimicking their potential nasal administration, whereas SEM studies provide a deeper understanding of the actual morphology of the generated formations. For example, the previously mentioned 100 nm joint particles (Figure 6/γ) might have aggregated even before the SMPS measurements, meaning that SMPS probably assigned a larger effective size to this group of particles. However, based on the SEM pictures, we can ascertain that joint particles have a high surface to volume ratio, which is favorable for increasing the dissolution rate of drugs.

For the SMPS analysis, the ablated particles are immediately transferred to the detector. However, the measured data are partially influenced by the transfer function of the SMPS system, and only a small fraction of the 100 nm or smaller particles can reach the detector due to collisions. Additionally, particles over 1 µm might be lost due to sedimentation. SMPS measurements cannot separate previously aggregated particles, thus they determine an effective diameter of the aggregate. On the other hand, the particles collected on the filter for SEM may undergo thermal, mechanical or chemical interactions with one another or with the environment. For example, they can undergo thermal relaxation and crystallization, and relatively smaller particles may join in the air to create larger structures. Based on the SEM images, it cannot be concluded if single-particle or previously aggregated chain particle groups were collected on the filter.

In case of PLA, the three main types of particles (as presented in Section 3.2.1 SEM and in Figure 6) are thought to have formed differently.

The smooth spheres (Figure 6/β) might be molten and re-solidified particles. After melting, the spherical shape was favorable, and due to fast congealing, the crystalline lattice arrangement could not be achieved. In the case of the lamella-covered spheres, we suppose that crystallization started in the outer regions, and the block-like crystallites started to peel off the spheres, resulting in these “bushy”/”pineal” spheres (Figure 6/α). This morphology shows a resemblance to spray-dried particles [33,34,35], but the PLA-generated particles were smaller, and no additive was needed.

The little spherical particles forming a web-like structure (Figure 6/γ) could have aggregated from previously melted droplets, or condensed from the gas phase. Femtosecond laser ablation-generated silicon and graphite nanoparticles were thoroughly investigated, and the mechanism was divided into several states: surface melting, transition into an overcritical fluid, evaporation, plume formation, critical vapor supersaturation, nanoparticle formation via condensation, and finally bonding nanoparticles creating web-like aggregates [36]. In the case of carbonaceous aerosol particles generated by laser ablation, fractal aggregates were described [37], and these are similar to the web-like structures on our SEM images. That study suggests that the formation of such particles can be described by a three-step simplified model [38]: nucleation, coagulation and aggregation, driven by favorable energy conditions. In the laser ablation-created plasma state, the atoms can collide, creating nanoparticle nuclei. These primary particles then collide, creating bigger spheres until the surface to volume ratio reaches an ideal value. After this, fractal aggregates appear as weaker bonds form between the colliding particles.

Comparing the above model for atomic nanoparticle formation with our observations, we suppose that it may apply to meloxicam too. However, since meloxicam is a heat-sensitive organic compound, it is reasonable to further investigate the phase changes of meloxicam without chemical damage. Databases state that the melting point of meloxicam is 254 °C, and it also decomposes at this temperature [30]. The temperature of the plume most likely exceeds this point, but the particles investigated by FTIR (Figure 3) and Raman spectroscopy (Figure 4) showed no chemical decomposition of meloxicam. Presumably, the decomposition products came into being in volatile form, leaving no measurable residue. Alternatively, it is also possible that the temperature and pressure conditions in the plume fall in a part of the phase diagram where phase transition occurs, without reaching the decomposition temperature. It must be noted that during femtosecond ablation, the energy transfer and plume formation occur on a much shorter time scale than molecular bond breaking. Unfortunately, we found no information on the temporal dynamics of the thermal decomposition of meloxicam.

On the other hand, our observations indicate that particle formation during PLAL is caused by mechanical rather than thermal effects. The laminar, block-like and needle-shaped particles can be fractures of the pressed tablet, which preserved their solid state and retained the crystal structure (Figure 7). The formation mechanism may be similar to that in a previously published experiment investigating nanosecond ablation in liquid [24]. In the current experiment, femtosecond laser irradiation induced fast material decomposition in a thin surface layer of the target, and the explosive expansion of the plasma plume that was created resulted in the formation of a shock wave. Then, as the propagating shock wave reached an intact surface region, mechanical spallation and material ejection occurred. The rounded particle edges seen in the SEM images might be due to thermal effects or recrystallization processes.

## 5. Conclusions

The laser ablation of meloxicam both in air and in distilled water has proven to be a successful method to generate size-reduced drug particles without inducing any chemical change in the solid phase, as confirmed by FTIR and Raman spectroscopy. Significant size reduction was achieved at various laser fluences without any additives. When compared to the 10–50 µm average particle size of the original meloxicam powder, the PLA-generated particles were mainly 100 nm or 1–2 µm in size, while the PLAL-produced fractures measured a few µm.

However, PLA and PLAL resulted in quite different particle morphologies. PLA mainly yielded spheres of different sizes. The most abundant forms were 100 nm spherical particles, creating nanoaggregates, and building up a web-like structure. Nanoaggregate production by laser ablation has previously been described only in cases of elemental materials. To the best of our knowledge, this experiment was the first to deal with the PLA of a complex molecular structure.

The PLAL-generated particles were mostly fractures or residues of surface spallation, supposedly induced by shock waves.

Based on our results, laser ablation is a suitable method for the particle size reduction of meloxicam particles, in order to reach the nano- and micrometer size range. Depending on the media, different morphologies can be obtained. The attainable submicron size range of the generated particles predicts that PLA and PLAL can become the preferred techniques for the preparation of innovative products for pulmonary and nasal drug administration via inhalation. The pulmonary administration of meloxicam can be used to treat diseases involving lung inflammation, and the nasal application may be important in rapid analgesia. The high surface to volume ratio of the ablated meloxicam particles is also desirable for improving their bioavailability.

## Data Availability

Data available on request due to restrictions.
